# Altered Anesthetic Sensitivity of Mice Lacking *Ndufs4*, a Subunit of Mitochondrial Complex I

**DOI:** 10.1371/journal.pone.0042904

**Published:** 2012-08-17

**Authors:** Albert Quintana, Philip G. Morgan, Shane E. Kruse, Richard D. Palmiter, Margaret M. Sedensky

**Affiliations:** 1 Howard Hughes Medical Institute and Department of Biochemistry, University of Washington, Seattle, Washington, United States of America; 2 Department of Anesthesiology and Pain Medicine, University of Washington and Center for Developmental Therapeutics, Seattle Children's Research Institute, Seattle, Washington, United States of America; Massachusetts General Hospital, United States of America

## Abstract

Anesthetics are in routine use, yet the mechanisms underlying their function are incompletely understood. Studies *in vitro* demonstrate that both GABA_A_ and NMDA receptors are modulated by anesthetics, but whole animal models have not supported the role of these receptors as sole effectors of general anesthesia. Findings in *C. elegans* and in children reveal that defects in mitochondrial complex I can cause hypersensitivity to volatile anesthetics. Here, we tested a knockout (KO) mouse with reduced complex I function due to inactivation of the *Ndufs4* gene, which encodes one of the subunits of complex I. We tested these KO mice with two volatile and two non-volatile anesthetics. KO and wild-type (WT) mice were anesthetized with isoflurane, halothane, propofol or ketamine at post-natal (PN) days 23 to 27, and tested for loss of response to tail clamp (isoflurane and halothane) or loss of righting reflex (propofol and ketamine). KO mice were 2.5 - to 3-fold more sensitive to isoflurane and halothane than WT mice. KO mice were 2-fold more sensitive to propofol but resistant to ketamine. These changes in anesthetic sensitivity are the largest recorded in a mammal.

## Introduction

The molecular mechanisms responsible for the effects of volatile anesthetics are far from clear. Although volatile anesthetics inhibit excitatory synaptic transmission and enhance inhibitory signaling, there is little agreement as to how this phenomenon occurs [1]–[3]. Ligand-gated ion channels initially emerged as the leading candidates to mediate these effects. Both GABA_A_ and NMDA receptors were initially viewed as likely volatile anesthetic targets, by virtue of their physiologic functions and anatomic locations within the central nervous system (CNS) [3]. A large number of compelling *in vitro* studies substantiated these hypotheses, since volatile anesthetics could potentiate inhibitory currents through GABA_A_ channels, or inhibit excitatory transmission in glutamatergic neurons [1], [4]. However, for a number of different possible reasons, whole animal models have not supported the hypothesis that NMDA and GABA_A_ receptors mediate all aspects of general anesthesia produced by volatile anesthetics [5], [6]. To date the largest change in a mammal to a volatile anesthetic is a 40% decrease in sensitivity to halothane in a mouse that lacks a 2-pore potassium channel, TREK-1 [7].

In a forward genetic screen in the nematode, *C. elegans*, we identified a mutation, *gas-1(fc21)*, that caused a very significant hypersensitivity to all volatile anesthetics [8]. The *gas-1* gene encodes a highly conserved subunit of complex I of the electron transport chain (83% similar to the human orthologue NDUFS2) [9], [10]. RNAi inhibition of most complex I subunits also increased volatile anesthetic sensitivity [11]. Interestingly, mutations in subunits of complex II, III, or IV did not change sensitivity of *C. elegans* to volatile anesthetics, even though animals carrying these mutations share many other phenotypes with *gas-1* [12], [13]. Children with defects in complex I function were hypersensitive to sevoflurane, whereas children with defects in other steps of electron transport within the mitochondrion were not, even though they were indistinguishable in symptoms of mitochondrial disease from the complex I-deficient patients [14]. Although the data obtained from patients predicted enhanced sensitivity of other mammals with complex I dysfunction, the sample size was low, the genes involved were unknown, and controls were a mixed population.

A mouse model with complex I deficiency was developed by conditional inactivation of the *Ndufs4* gene that encodes an 18 kD subunit of complex I. This subunit is not directly involved in electron transport, but appears to play a role in assembly or stability of the complex [15], [16]. Homozygous *Ndufs4*-null mice appear neurologically normal at weaning, but by post-natal day 35 (PN35) the KO mice manifest increasing ataxia, failure to thrive, and usually die by PN55. This strain has been established as a model for Leigh syndrome, the most common infantile mitochondrial disorder. Mice with selective loss of *Ndufs4* function within the CNS have the same behavioral phenotype as the total KO mice [16].

Initial attempts to anesthetize KO mice using standard conditions were often fatal. Those observations along with the knowledge of sensitivity of worms and children with complex I deficiencies prompted us to hypothesize that the KO animals would be hypersensitive to volatile anesthetics. We determined the sensitivity to anesthetics shortly after weaning (PN23 to 27), when the animals are still behaviorally normal and before there is any evidence of neuronal degeneration in the CNS [16]. Remarkably, KO mice displayed the greatest hypersensitivity to volatile anesthetics ever recorded for a mammal. This sensitivity extended to the non-volatile GABA_A_ facilitator and agonist, propofol [17], but not to the NMDA antagonist, ketamine [18], [19]. The differential sensitivity to different classes of anesthetics may provide a clue to the role of complex I in mediating anesthetic action.

## Methods

### Ethics Statement

This study was carried out in strict accordance with the recommendations in the Guide for the Care and Use of Laboratory Animals of the National Institutes of Health. All animal experiments were performed with the approval of the Animal Care and Use Committee of the University of Washington (IACUC #2183-02). No surgery was performed and all efforts were made to minimize suffering.

### Anesthetic sensitivity

Mice were maintained with rodent diet (5053, Picolab, Hubbard, OR) and water available *ad libitum* in a vivarium with a 12-h light-dark cycle at 22°C. The KO mice were generated by crosses of heterozygotes on a C57Bl/6 genetic background, genotyped by polymerase chain reaction at PN22; KO and WT littermate controls were tested for anesthetic sensitivity during the next week.

Mice were anesthetized with halothane or isoflurane and their temperature was maintained by radiant heat according to the techniques of Sonner [20], [21]. Animals were between 23–27 days old at the time of exposure to anesthetic. Failure to respond to a non-damaging tail clamp was recorded. Responses of the same mouse to different doses of the volatile anesthetics were measured after 15 min of equilibration between doses. All animals were exposed to a single anesthetic drug. Samples of isoflurane or halothane were taken at different delivery settings and measured by gas chromatography as described [8]. The non-volatile anesthetics: propofol (Diprivan®, AstraZeneca, Wilmington DE, USA) or ketamine (Ketaset®, Fort Dodge, IA, USA) were injected intra-peritoneally with drug at a concentration of 10 mcg/ul. Animals were tested for loss of righting reflex (LORR) at 5-min intervals following injection. Animals were kept warm on a heating pad throughout. Animals were allowed to recover for at least 24 h before testing again at a different dose of the same anesthetic. No animal received more than four test doses. Animals that did not lose righting reflex within 30 min of injection were denoted as a no LORR.

### Statistics

**Figure 1:**
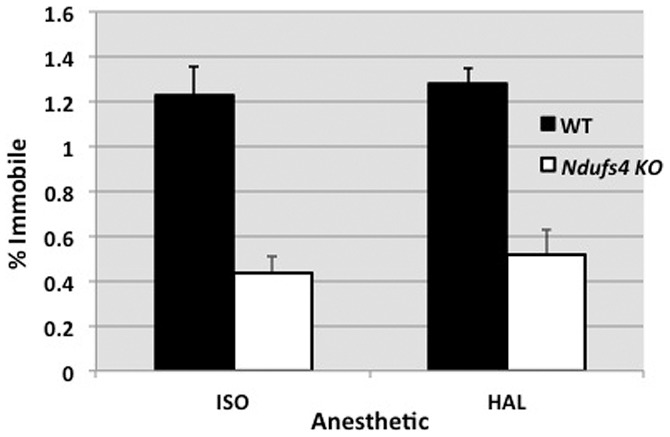
EC_50_s for isoflurane (ISO) and halothane (HAL) to cause immobility in response to tail pinch. Dark bars show the EC_50_s for wild-type (WT) mice (n = 10, ISO; n = 6, HAL); open bars show the values for the *Ndufs4* KO mice (n = 10, ISO; n = 6, HAL). Error bars show the standard deviations. The values for the KO animals were significantly different from those for WT with a p<0.01.

The effective concentration for 50% of the animals tested (EC_50_) for volatile anesthetics was determined as described by Sonner *et al.*, using an up and down method [20]. The effective dose for 50% of maximum effect (ED_50_) values for propofol and ketamine were determined by constructing a dose-response curve for each drug and taking the midpoint of the curve. Values for EC_50_s and ED_50_s were compared between the WT and KO strains using GraphPad Prism® and Sigmaplot® software (T-test analysis for EC_50_ and built-in dose-response curve fitting for ED_50_) with a modification as described by Waud [22]. Values for EC_50_ and ED_50_ were compared between the WT and KO strains using a Students t-test. Significance was defined as a p<0.01. Error bars in Figure 1 represent the standard deviations of the mean. Errors for propofol and ketamine represent Standard Errors of the mean.

## Results

*Ndufs4* KO mice were extremely hypersensitive to isoflurane (Figure 1), with an EC_50_ that was about one third that of their WT littermates (KO EC_50_ = 0.44±0.07%; WT EC_50_ = 1.23±0.13%). KO mice were also hypersensitive to halothane (KO EC_50_ = 0.52±0.11%; WT EC_50_ = 1.28±0.07%, WT) as shown in Figure 1. No animals displayed any seizure-like activity with exposure to the volatile anesthetics. Animals reached steady state for their response within 5 min of volatile anesthetic exposure and they recovered from exposure to the gases within 15 min of breathing room air. KO and WT mice displayed vigorous responses to tail pinch in air and at sub-anesthetics doses of volatile anesthetics. The EC_50_ values of WT mice were similar to that previously reported for the C57Bl/6 strain [20]. Animals lost righting reflex at concentrations too low to be delivered with standard vaporizers.

**Figure 2:**
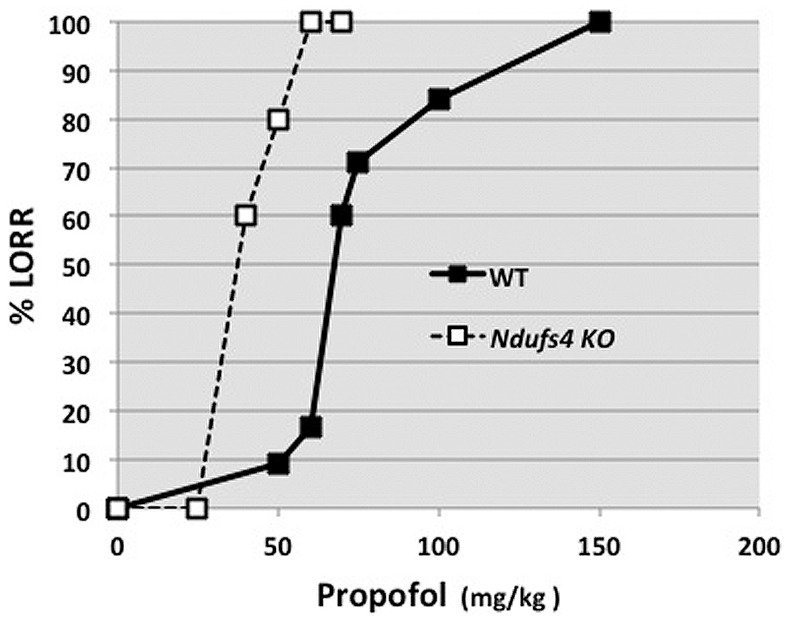
The dose response curves for WT and *Ndufs4* KO mice for LORR after intraperitoneal propofol. Dose-response curves were generated using the percentage of mice that presented LORR at the measured concentrations (n = 5–7 per group for each injection dose). The values for the KO animals were significantly different from those for WT (p<0.01).

KO mice were also hypersensitive to propofol, although the shift was not as extreme as that for the volatile agents (Figure 2). The dose of propofol that produced LORR in the KO mice was about one half that of their WT littermates (KO ED_50_ = 38±5 mg/kg; WT ED_50_ = 67±6 mg/kg). The maximum effect of propofol was observed within 5 min of injection in both the WT and KO mice and all animals recovered righting reflex within 15 min of injection. The ED_50_ for propofol in WT mice agrees with published data [23], [24].

**Figure 3:**
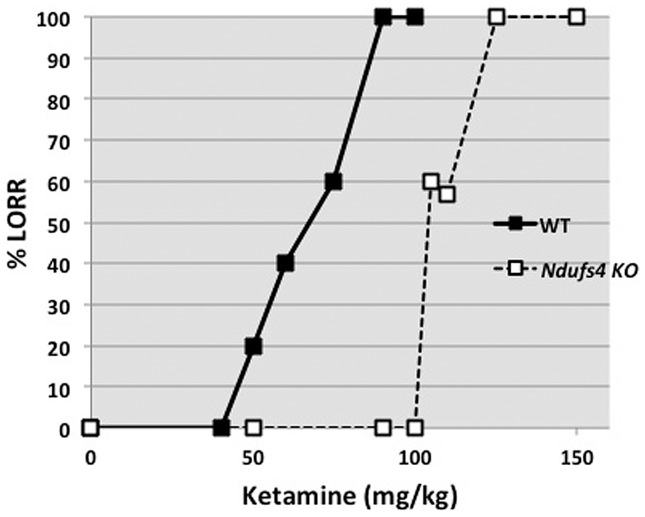
The dose response curves for WT and *Ndufs4* KO mice for LORR after given intraperitoneal ketamine. Dose-response curves were generated using the percentage of mice that presented LORR at the measured concentrations (n = 5–7 per group for each injection dose). The values for the KO animals were significantly different from those for WT (p<0.01).

In contrast to both the previous results, the KO animals were strikingly resistant to the effects of ketamine (Figure 3), and were significantly resistant to the LORR (KO ED_50_ = 106±5 mg/kg; WT ED_50_ = 69±4 mg/kg). The maximum effect of ketamine on LORR was seen within 5 min of injection for both WT and KO animals and all animals recovered by 15 min after injection. The ketamine data for the WT animals agree with a published value of 65 mg/kg [23], [24]. Recovery times for all drugs were similar between WT and KO animals.

## Discussion

We report here the largest change in sensitivity to volatile anesthetics recorded for a mammal. The ability of complex I mutations to change response to volatile anesthetics transcends many phyla, which implies an ancient, common mechanism of action. The KO mice were equally hypersensitive to two volatile anesthetics that are quite different in structure. These results are in contrast to our results with mutations in transmembrane leak channels that result in differential sensitivity to isoflurane and halothane in *C. elegans* [25]. As we have noted previously, we believe that mitochondrial defects affect a downstream target relative to the leak channels, such that sensitivities to all volatile anesthetics are affected [26].

Isoflurane and halothane both enhance GABA_A_ receptor signaling while antagonizing NMDA receptor signaling [17] although it is not clear that these effects cause the anesthetized state. Numerous structure-function experiments have shown that GABA_A_ receptors are targets of volatile anesthetics, using *in vitro* assays. However, when putative targets that were thought to be resistant to anesthetic action were tested in genetically engineered mice, the responses to volatile anesthetics were insufficiently affected [5], [27]–[29]. Thus, the true target(s) of volatile anesthetics remain enigmatic.

The degree/direction of changes in sensitivity of *Ndusf4(KO)* is not uniform across different classes of anesthetic drugs. The KO mice were also hypersensitive to propofol, which is known to act primarily on GABA_A_ receptors [17]. However, the hypersensitivity was not as great as with the volatile anesthetics. In addition, any explanation for the hypersensitivity of the KO animals to volatile agents and to propofol must also account for the surprising resistance of these animals to an NMDA antagonist, ketamine [18], [19]. Since times of onset and recovery for propofol and ketamine were the same for WT and KO animals, pharmacokinetics did not play a major role in these responses; the responses represent changes in pharmacodynamics. The observation that the KO mice are resistant to ketamine argues against the possibility that the KO mice manifest a general neuronal dysfunction at the time of testing that makes them hypersensitive to all neuronal depressants. This is in agreement with studies in a mouse model of Alzheimer's disease, which also demonstrated no increase in anesthetic sensitivity despite generalized CNS depression. [30], [31]. It also indicates that the targets that produce the anesthetic state are not identical between ketamine and the other anesthetics tested here. Ketamine anesthetic action may be unique, as it has been suggested to involve increased activation and cortical synchronization rather than neuronal inactivation [32]. Inhibition of HCN1 channels has also been recently suggested as a contributing factor in the hypnotic actions of ketamine further indicating that ketamine function is more complicated than usually thought [33]. The resistance to ketamine in these mice raises an intriguing question as to whether similar changes might be present in humans and may suggest future studies.

How can mitochondrial dysfunction cause extreme hypersensitivity to volatile anesthetics and propofol, and why are defects in complex I function so important? Complex I is responsible for over half of the electron transport necessary to generate the mitochondrial membrane potential and drive ATP synthesis [34]. The mitochondrial TCA cycle generates glutamate and the precursors of GABA and, while a small part of total energy requirements, the glutamate/glutamine cycle between neurons and glia is dependent on glycolysis and oxidative phosphorylation [35]. Complex I also has the potential to generate reactive oxygen species (ROS), which can result in deleterious oxidation events and/or serve as a critical signaling molecule, when not functioning optimally [34]. Thus, there are many possible ways that loss of complex I might cause hypersensitivity to volatile anesthetics. Most notable is the finding that presynaptic function in glutamatergic neurons is extremely sensitive to complex I function [36].

It is possible that anesthetic sensitivity of the KO mice (as well as *C. elegans* and children) with complex I deficiency is due to the direct actions of these compounds on defective complex I [37], further inhibiting its activity and resulting in the inability to maintain ATP production and/or essential signaling necessary to maintain neuronal activity. Xi *et al.* [37] noted that several mitochondrial proteins bind halothane, including three from complex I, consistent with the possibility that complex I may be a direct anesthetic target. In both worms and in mammals, movement of electrons through complex I is clearly the most sensitive step within the mitochondrial respiratory chain to disruption by volatile anesthetics [9], [38], [39] whereas defects in respiratory complexes II, III, and IV do not affect anesthetic sensitivity [12]–[14], [40].

An alternative idea is that anesthetics act primarily on ion channels as is generally hypothesized [6], [17], [35], but select populations of neurons within the CNS may depend on optimal complex I function to maintain neuronal activity [34]. Assuming that volatile anesthetics work by altering synaptic transmission in some specific areas of the CNS, it may be that the animal is able to match ATP supply to demand in most of the CNS, but has insufficient ATP to support synaptic transmission by some crucial neurons. Thus, if those neurons were already compromised due to complex I mutations and consequently functioning at maximum capacity, then modulation of ion currents by anesthetics could selectively compromise their ability to function adequately.

Although many authors hypothesize that anesthetics act diffusely throughout the CNS, others attribute their actions to specific brain regions; for example, a portion of the rat brainstem has been dubbed the “mesopontine tegmental anesthesia area” since injection of GABA_A_ receptor agonists into this region produces anesthesia [41]–[44]. The central medial thalamic region of the rat brain has also been shown to be as crucial to maintaining consciousness [44]. However, considerable controversy still surrounds the putative location in the brain for producing the anesthetic state. The KO mice display progressive gliosis and eventually neurodegeneration in specific brain regions; primarily the olfactory bulb, vestibular nucleus, posterior lobes of the cerebellum and deep cerebellar nuclei. However, some other brain regions are without obvious gliosis, for example, the pre-Bötzinger complex, yet are also affected by the mitochondrial defect [16], [45]. Consequently, we assume that *Ndfus4* deficiency does not affect all neurons equally. The differences in sensitivity to loss of *Ndufs4* could be attributable to differences in (a) intrinsic activity of the neurons, (b) extent of activation in response to changing conditions, or (c) regulation of complex I, for example, by phosphorylation.

It may be possible to identify brain region(s) and neuronal type(s) where *Ndufs4* functions to maintain anesthetic sensitivity. Because the *Ndufs4* allele in our KO mice can be inactivated by Cre recombinase, it is possible to use Cre-expressing viruses or specific Cre-driver lines of mice to selectively inactivate *Ndufs4* in specific cell types or brain regions. Alternatively, it is possible to restore *Ndufs4* function to specific cells or brain regions in KO mice. These approaches were used to demonstrate that the vestibular nucleus of KO mice is selectively compromised leading to fatal breathing abnormalities [45]. An additional challenge will be to ascertain whether complex I dysfunction indirectly facilitates anesthetic sensitivity or whether volatile anesthetics interfere with complex I function to directly control sensitivity.
